# *Riemerella anatipestifer* GldM is required for bacterial gliding motility, protein secretion, and virulence

**DOI:** 10.1186/s13567-019-0660-0

**Published:** 2019-06-04

**Authors:** Zongchao Chen, Xiaolan Wang, Xiaomei Ren, Wenlong Han, Kanwar Kumar Malhi, Chan Ding, Shengqing Yu

**Affiliations:** 1 Shanghai Veterinary Research Institute, Chinese Academy of Agricultural Sciences (CAAS), Shanghai, China; 2 Jiangsu Agri-animal Husbandry Vocational College, Veterinary Bio-pharmaceutical, Jiangsu Key Laboratory for High-Tech Research and Development of Veterinary Biopharmaceuticals, Taizhou, Jiangsu China

## Abstract

*Riemerella anatipestifer* is a major pathogenic agent of duck septicemic and exudative diseases. Genetic analyses suggest that this pathogen has a novel protein secretion system, known as the “type IX secretion system” (T9SS). We previously reported that deletion of the *AS87_RS08465* gene significantly reduced the bacterial virulence of the *R. anatipestifer* strain Yb2, but the mechanism remained unclear. The *AS87_RS08465* gene is predicted to encode the gliding motility protein GldM (GldM) protein, a key component of the T9SS complex. In this study, Western blotting analysis demonstrated that *R. anatipestifer* GldM was localized to the cytomembrane. Further study revealed that the adhesion and invasion capacities of the mutant strain RA2281 (designated Yb2Δ*gldM*) in Vero cells and the bacterial loads in the blood of infected ducks were significantly reduced. RNA-Seq and PCR analyses showed that six genes were upregulated and five genes were downregulated in the mutant strain Yb2Δ*gldM* and that these genes were mainly involved in the secretion of proteins. Yb2Δ*gldM* was also found to be defective in gliding motility and protein secretion. Liquid chromatography–tandem mass spectrometry analysis revealed that nine of the proteins had a conserved T9SS C-terminal domain and were differentially secreted by Yb2Δ*gldM* compared to Yb2. The complementation strain cYb2Δ*gldM* recovered the adhesion and invasion capacities in Vero cells and the bacterial loads in the blood of infected ducks as well as the bacterial gliding motility and most protein secretion in the mutant strain Yb2Δ*gldM* to the levels of the wild-type strain Yb2. Taken together, these results indicate that *R. anatipestifer* GldM is associated with T9SS and is important in bacterial virulence.

**Electronic supplementary material:**

The online version of this article (10.1186/s13567-019-0660-0) contains supplementary material, which is available to authorized users.

## Introduction

*Riemerella anatipestifer* is a causative pathogen of diseases in ducks, geese, turkeys, and various other domestic and wild birds [1]. Infected ducks show clinical signs of lethargy, diarrhea, and respiratory and nervous symptoms, all of which cause serious economic losses in the duck industry [2]. Several virulence factors of *R. anatipestifer* have been identified, including CAMP cohemolysin, OmpA, glycosyl transferase, nicotinamidase PncA, VapD and other factors associated with lipopolysaccharide synthesis and iron acquisition [2–10]. However, the mechanisms of *R. anatipestifer* virulence are not completely understood, which hinders the development of an efficient strategy to control this disease.

*Riemerella anatipestifer* is a member of the phylum *Bacteroidetes*. A novel protein secretion system, known as the “type IX secretion systems” (T9SSs) or “Por secretion system”, has recently been frequently found in members of the phylum *Bacteroidetes* [11, 12]. Many virulence factors of pathogenic bacteria are either secreted proteins or the secretion systems themselves [13]. T9SS is associated with bacterial gliding motility and protein secretion and is considered to be a virulence factor in many pathogens [14, 15]. Genetic analyses have shown that GldK, GldL, GldM, GldN, SprA, SprE, SprT, PorU, and PorV are components of T9SS in *Flavobacterium johnsoni* [16–19]. The proteins secreted by T9SSs have a typical N-terminal signal peptide and traverse the cytoplasmic membrane into the periplasm via the general secretion (Sec) system. The proteins also typically have conserved C-terminal domains (CTDs) that target them to T9SS for secretion across the outer membrane [20]. SprT, encoded by the *sprT* gene, is a T9SS protein involved in protein secretion in *R. anatipestifer*. T9SS is functional in *R. anatipestifer* and contributes to its virulence by exporting key proteins [21]. The T9SS component GldM is required for bacterial gliding motility and the secretion of the cell-surface motility adhesins SprB and RemA in *F. johnsoniae* [22]. GldM is also required for the secretion of ChiA, which digests colloidal chitin and many other proteins [17, 18, 23]. Sequence analyses have shown that *R. anatipestifer* GldM has a single predicted transmembrane helix near the N-terminus that is highly conserved, suggesting that it functions beyond simple membrane anchoring, possibly in protein secretion and/or bacterial motility [19, 24].

Genetic techniques developed for *R. anatipestifer* have been used to identify many virulence genes [25]. We previously generated a virulence-attenuated mutant of strain Yb2, in which the Tn*4351* transposon was inserted into the *AS87_08795* gene. In this study, this mutant strain was shown to be defective in gliding motility and protein secretion. We also demonstrated that the *AS87_08795* gene encodes a T9SS component that is involved in the virulence of *R. anatipestifer.*

## Materials and methods

### Ethics statement

The study protocol was approved by the Institutional Animal Care and Use Committee of Shanghai Veterinary Research Institute, the Chinese Academy of Agricultural Sciences (approval no. Shvri-po-2017090877), and was conducted in strict accordance with the recommendations outlined in the Guide for the Care and Use of Laboratory Animals. One-day-old Cherry Valley ducks were obtained from Zhuang Hang Duck Farm (Shanghai, China) and housed in cages at a controlled temperature of 28–30 °C under biosafety conditions, with water and food provided ad libitum.

### Bacterial strains, plasmids, and culture conditions

The bacterial strains and plasmids used in this study are listed in Table 1. *R. anatipestifer* serotype 2 strain Yb2 is the wild-type strain. The *R. anatipestifer* strains were grown at 37 °C in tryptic soy broth medium (TSB, Difco, Franklin Lakes, NJ, USA). To prepare the solid tryptic soy agar (TSA) medium, 1.5 g of agar was added to 100 mL of TSB. The *Escherichia coli*–*F. johnsoniae* shuttle plasmid pCP29 and *E. coli* strain S17-1 were kindly provided by Mark J. McBride (University of Wisconsin–Milwaukee, Milwaukee, WI, USA). The *E. coli* strains were grown at 37 °C on Luria–Bertani (LB) plates or in LB broth. Antibiotics were used at the following concentrations when required, unless otherwise indicated: ampicillin (100 μg/mL), chloramphenicol (30 μg/mL), erythromycin (0.5 μg/mL), kanamycin (50 μg/mL), streptomycin (50 μg/mL), and cefoxitin (5 μg/mL).

**Table 1:** **Strains, plasmids, and primers used in this study**

Strains, plasmids, primers	Characteristics
Yb2	*Riemerella anatipestifer* serotype 2 strain
*Escherichia coli* S17-1	*λ*pirhsdR pro thi; chromosomally integrated RP4-2, Tc::Mu Km::Tn7
Yb2Δ*gldM*	Tn*4351* insertion mutant of *R. anatipestifer* Yb2, *gldM*::Tn
cYb2Δ*gldM*	Mutant Yb2Δ*gldM* carrying plasmid pCP29-*gldM*
pCP29	ColE1 ori (pCP1 ori), Ap^r^ (Em^r^); *E. coli*–*F. johnsoniae* shuttle plasmid
pCP29-*gldM*	pCP29 containing *ompA* promoter and *gldM* ORF, *cfxA*(Ap^r^)
Primers	
AS87_RS08465-F	5′-ATGGCAAAGGAAAAATT-3′
AS87_RS08465-R	5′-CTGAACATTTATCACTACTGGAG-3′
*ompA* promoter P1	5′-CAGGTACCAGCTAAAATTTTGGCAGTAAC-3′ (*Kpn*I site underlined)
*ompA* promoter P2	5′-CGACTCGAGCATTCCAATTCTCTTATTATC-3′ (*Xho*I site underlined)
gldM-orf-F	5′-CAGGTACCATGGCAAAGGAAAAATT-3′ (*Kpn*I site underlined)
gldM-orf-R	5′-CGACTCGAGCTGAACATTTATCACTACTGGA-3′ (*Xho*I site underlined)
gldM-pro-F	5′-CGGGATCCATTATACGCTCTTACAATGATAC-3′ (*Bam*HI site underlined)
gldM-pro-R	5′-GCGTCGACCTGAACATTTATCACTACTGGAG-3′ (*Sal*I site underlined)
RA ldh-F	5′-ATGAATTATTTTAAACTGCT-3′
RA ldh-R	5′-TTAGTCTAATTTCTGTATAT-3′
RA 16S rRNA-F	5′-TCTAAAATGAGATGTTCCA-3′
RA 16S rRNA-R	5′-ACGAAAGCGTGGGGAGTGG-3′
Tn4351-F	5′-TGGCACCTTTGTGGTTCTTAC-3′
Tn4351-R	5′-GAGAGACAATGTCCCCCTTTC-3′

ORF: open reading frame, *cfxA:* cefoxitin-resistance gene.

### Construction of the mutant strain Yb2Δ*gldM* and complementation strain cYb2Δ*gldM*

The mutant strain Yb2Δ*gldM* was constructed by inserting the Tn4351 transposon into the *AS87_08795* gene of the wild-type strain Yb2 (which was designated the *AS87*-*RS08465* gene). Polymerase chain reaction (PCR) was used to identify the wild-type strain Yb2 and mutant strain Yb2Δ*gldM* with the primers 16S rRNA-F/16S rRNA-R and AS87_RS08465-F/AS87_RS08465-R, respectively (Table 1). Inverse PCR was used to determine the insertion site of the transposon in the mutant strain. Briefly, genomic DNA of the mutant strain was digested with *Hin*dIII and ligated to form a closed circle. DNA adjacent to the insertion site was amplified with the Tn*4351*-specific primers TN4351-F/TN4351-R. DNA sequencing data were compared to a database using a BLAST search at the National Center for Biotechnology Information website. The polar effect of the mutation was determined by qPCR analysis of the adjacent gene’s expression.

The shuttle plasmid pCP29, which carries the *ompA* promoter of *R. anatipestifer*, was used to construct the complementation strain cYb2Δ*gldM* as described previously [26]. Briefly, the open reading frame (ORF) of *gldM* was amplified from *R. anatipestifer* Yb2 genomic DNA with the primers gldM-orf-F/gldM-orf-R, digested with *Kpn*I and *Xho*I, and ligated into pCP29 that had been digested with the same enzymes, generating the pCP29–*gldM* plasmid. The pCP29–*gldM* plasmid was then transferred into the mutant strain Yb2Δ*gldM* by conjugation to construct the complementation strain cYb2Δ*gldM.*

### Protein expression and antibody production

A 1464-bp fragment encoding the extramembranous part of GldM was amplified from the *R. anatipestifer* Yb2 genome with the primers GldM-pro-F/GldM-pro-R and ligated into the pET-30a(+) vector at the *Bam*HI and *Sal*I cloning sites. The resulting plasmid, pET–*gldM*, was sequenced and confirmed to be identical to the *R. anatipestifer gldM* sequence in the GenBank database (accession number AS87_RS08465). Competent *E. coli* strain BL21(DE3) cells were transformed with the recombinant plasmid pET–*gldM* to express the protein. The transformed *E. coli* cells were cultured, and recombinant GldM protein (rGldM) expression was induced with 1 mM isopropyl β-d-1-thiogalactopyranoside for 6 h at 37 °C with shaking. The cells were harvested by centrifugation at 10 000 × *g* for 5 min at 4 °C, resuspended in lysis buffer (20 mM Na_3_PO_4_, 0.5 M NaCl, pH 7.4), and purified with HisTrap affinity columns (GE Healthcare, Uppsala, Sweden) according to the manufacturer’s protocol. Aliquots of the fractions obtained were analyzed via sodium dodecyl sulfate-polyacrylamide gel electrophoresis (SDS-PAGE). The protein concentrations were measured with a BCA protein assay kit (Beyotime, Shanghai, China), with bovine serum albumin (BSA) as the standard.

Two 2-month-old New Zealand rabbits were immunized three times with purified *R. anatipestifer* rGldM at 2-week intervals at a dose of 1 mg of purified rGldM in the same volume of Montanide ISA 50 V adjuvant (SEPPIC, Paris, France). The preimmune and postimmune sera were tested with an indirect enzyme-linked immunosorbent assay (ELISA) for rGldM to confirm the presence of anti-GldM antibodies. Qualified antiserum was screened via Western blotting.

### Western blotting analysis

#### Identification of cYb2ΔgldM by Western blotting

To determine whether the complementation strain cYb2Δ*gldM* expressed GldM, whole-cell proteins of the wild-type strain Yb2, mutant strain Yb2Δ*gldM*, and complementation strain cYb2Δ*gldM* were extracted and separated by SDS-PAGE and then electrophoretically transferred onto nitrocellulose (NC) membranes (Millipore, Billerica, MA, USA). The membranes were blocked in phosphate-buffered saline (PBS) containing 5% nonfat milk, washed with PBS containing 0.05% Tween 20, and incubated overnight with the rabbit anti-rGldM polyclonal antibody. A horseradish peroxidase-conjugated goat anti-rabbit IgG polyclonal antibody (Bio-Rad Laboratories, Hercules, CA, USA) was then applied, and the specific bands were developed with the Basic Luminol Chemiluminescent Kit (S-Wb001), visualized using a Tanon 5200 automatic chemiluminescence image analysis system (Tanon, Shanghai, China), and quantified using ImageJ software (National Institutes of Health, Rockville, USA). A rabbit anti-TonB-dependent receptor antibody was used as the control for protein loading. The intensities of the protein bands were analyzed with Quantity One software (Bio-Rad Laboratories).

#### Subcellular localization of GldM

The cytoplasmic proteins and membrane proteins from the wild-type strain Yb2 were fractioned with a bacterial membrane protein extraction kit (BestBio, Shanghai, China) according to the manufacturer’s protocol. The protein concentrations were determined with a BCA protein assay kit (Beyotime), with BSA as the standard. For Western blotting analysis, the subcellular fractions were separated by SDS-PAGE and then transferred onto a NC membrane. Western blotting was performed as described above. The rabbit anti-TonB-dependent receptor antibody was used as the control for protein loading.

### Gliding motility assay

The *R. anatipestifer* wild-type strain Yb2, mutant strain Yb2Δ*gldM*, and complementation strain cYb2Δ*gldM* were examined for movement over agar surfaces as previously described, with some modifications [27, 28]. Briefly, each strain was grown on TSA plates for 12 h, washed with TSB, and diluted to 2.5 × 10^3^ colony forming units (CFU)/mL. Aliquots (50 μL) of the cultures were plated onto TSB medium containing 0.5% agar. The colonies were grown for 24 h at 37 °C and examined by phase-contrast microscopy (Nikon D-Eclipse C1, Japan).

### Measurement of protease activity

The proteolytic activity of each strain was quantified as described previously [29] with modifications. In brief, the strains Yb2, Yb2Δ*gldM*, and cYb2Δ*gldM* were grown on TSA plates for 12 h; washed with sterile PBS; and adjusted to 2.5 × 10^9^ CFU/mL. These strains were then used to inoculate 5 mL volumes of ADCF-mAb medium (Hyclone) and incubated for 8 h at 37 °C with shaking at 220 rpm. The cultures were centrifuged at 19 950 × *g* for 10 min, and the supernatants were purified by passage through 0.45 µM HT Tuffryn syringe filters (PALL Life Sciences, Ann Arbor, MI, USA). The bacterial pellets were dried in an 80 °C heat block for 3 h, and the dry weights of the cell pellets were measured to calculate the proteolytic activity. A 2% azocasein (Sigma) solution was prepared in 0.05 M Tris–HCl (pH 7.4). The cell-free supernatant (50 µL) was mixed with 50 µL of azocasein substrate and incubated at 37 °C for 3 h. Triplicate assays were performed for each supernatant sample and negative controls (50 µL of ADCF-mAb medium). After incubation, 130 µL of 10% trichloroacetic acid was added to each sample, mixed, allowed to stand for 10 min at room temperature, and centrifuged at 19 950 ×* g* for 20 min to remove precipitated azocasein. An aliquot (100 µL) of the soluble supernatant was added to a flat-bottomed 96-well plate, and 200 µL of 1 M NaOH was added and mixed. The optical density at a wavelength of 450 nm (OD_450_) was determined with an iMark Microplate Absorbance Reader (Bio-Rad Laboratories). The raw optical density values at 450 nm (OD_450_) obtained from the triplicate assays were averaged for each supernatant sample, and the mean negative control OD_450_ was subtracted from these values. The proteolytic activity per milligram of dry cells was calculated as ([mean sample OD_450_ − mean negative control OD_450_]) × 1000 × 100/dry weight of the bacterial pellet.

### SDS-PAGE and liquid chromatography–tandem mass spectrometry (LC–MS/MS) analyses

The *R. anatipestifer* wild-type strain Yb2, mutant strain Yb2Δ*gldM* and complementation strain cYb2Δ*gldM* were grown in 200 mL of ADCF medium at 37 °C with shaking until OD_600_ reached 0.8. The cultures were centrifuged at 8000 × *g* for 10 min, and the supernatants were purified by passage through 0.22 μm polyvinylidene difluoride filters. The proteins in the supernatants were collected with 3-kDa Amicon Ultra Centrifugal Filter Units (Sigma) and stored at −80 °C until analysis. The protein quality was determined by SDS-PAGE, followed by Coomassie Brilliant Blue staining. The proteins were analyzed with LC–MS/MS as described previously [16]. The resulting MS/MS data were evaluated using a MASCOT search engine based on the UniProt database. The sequences of identified proteins were searched using the BLAST server to identify homologous sequences of *R. anatipestifer* Yb2 and their putative functions.

### RNA sequencing and differential expression analysis

Total RNA was isolated from the wild-type strain Yb2, mutant strain Yb2Δ*gldM* and complementation strain cYb2ΔgldM with TRIzol Reagent (Invitrogen, Carlsbad, CA, USA) according to the manufacturer’s instructions. Ribosomal RNA was removed with the Ribo-Zero Magnetic Gold Kit (Epicenter, USA), and Illumina RNA-Seq libraries were generated with the TruSeq SR Cluster Kit v3-cBot-HS (Illumina). The complete libraries were sequenced for 100 cycles on an Illumina HiSeq 2000 system according to the manufacturer’s instructions [2].

Low-quality reads and adaptors were removed from raw reads. Cleaned reads were aligned to the *R. anatipestifer* Yb2 genome using the RNASequel software HISAT (Version 2.0.1-beta) [30, 31]. Trimmed reads were aligned to the *R. anatipestifer* Yb2 genome with the TopHat2 software (version 2.0.9) [32]. The transcript levels were calculated as fragments per kilobase of cDNA per million fragments mapped. Differentially expressed genes with a fold change (cutoff) of 2.0 were detected with the Cufflinks software (version 2.1.1) [33] and were considered statistically significant if the fold change was > 2.0 and the *p* value was < 0.05.

### Real-time quantitative PCR (qPCR) analysis

qPCR was used to confirm the transcription levels of the differentially expressed genes identified in the RNA-Seq analysis. Gene-specific primers were designed with the Primer3 online software version 0.4.0 and are described in Additional file 1. The gene encoding l-lactate dehydrogenase (*ldh*) was used as the internal control, and its transcription was measured with the primers RA ldh-F/RA ldh-R (Table 1) [34, 35]. The RNA samples were extracted and purified as described above. cDNA was synthesized with PrimeScript RT Master Mix (Takara) according to the manufacturer’s protocol and diluted threefold as the template. qPCR was performed with GoTaq qPCR Master Mix (Promega, Fitchburg, WI, USA) using the following parameters: 95 °C for 2 min, 40 cycles of 95 °C for 15 s, 55 °C for 15 s and 68 °C for 20 s, followed by one cycle of 95 °C for 15 s, 60 °C for 15 s and 95 °C for 15 s. The reactions for each sample were performed in triplicate and run on a Mastercycler ep realplex4 apparatus (Eppendorf, Germany). The transcription levels were quantified with the 2^−ΔΔCt^ method [33].

### Bacterial adherence and invasion assays

Adhesion and invasion assays were performed on Vero cells (ATCC CCL-81) as previously described [2]. Briefly, Vero cells (approximately 2.5 × 10^5^ cells/well) were infected with the appropriate strain at a multiplicity of infection (MOI) of 50, incubated for 1.5 h at 37 °C under 5% CO_2_, rinsed three times with sterile PBS, and lysed with 0.1% trypsin (100 µL/well). The number of cell-adherent bacteria was determined after each lysate was serially diluted tenfold and spread onto a TSA plate. For the invasion assay, infected cells were incubated for an additional 1 h with Dulbecco’s modified Eagle’s medium supplemented with 100 µg/mL gentamicin to kill any extracellular bacteria. Infected cells were then washed three times with PBS and lysed, and the number of intracellular bacteria was determined. All the assays described here were performed in triplicate and replicated three times.

### Assessment of virulence in vivo

The median lethal dose (LD_50_) of the mutant strain Yb2Δ*gldM* was determined as described in our previous study [25]. To determine whether *R. anatipestifer gldM* is involved in the systemic invasion and dissemination of the bacterium, the bacterial loads in the blood of infected ducks were measured and analyzed. Briefly, 30 18-day-old Cherry Valley ducks were randomly divided into three groups (10 ducks per group) and intramuscularly infected with the wild-type strain Yb2, mutant strain Yb2Δ*gldM*, or complementation strain cYb2Δ*gldM* at a dose of 10^6^ CFU in 0.5 mL of PBS. Blood samples were collected at 6, 12, and 24 h post-infection (hpi), diluted appropriately, and plated on TSA for bacterial counting [2].

### Statistical analysis

Statistical analyses were conducted using GraphPad Software version 6.0 (La Jolla, CA, USA). One-way analysis of variance (ANOVA) was used for analyses of growth curves, protease activity, adhesion and invasion data, and two-tailed independent Student’s *t* test was used for analyses of the bacterial loads in blood. *p* values of < 0.05 were considered significant.

## Results

### Characterization of the mutant strain Yb2*ΔgldM* and complementation strain cYb2*ΔgldM*

**Figure 1:**
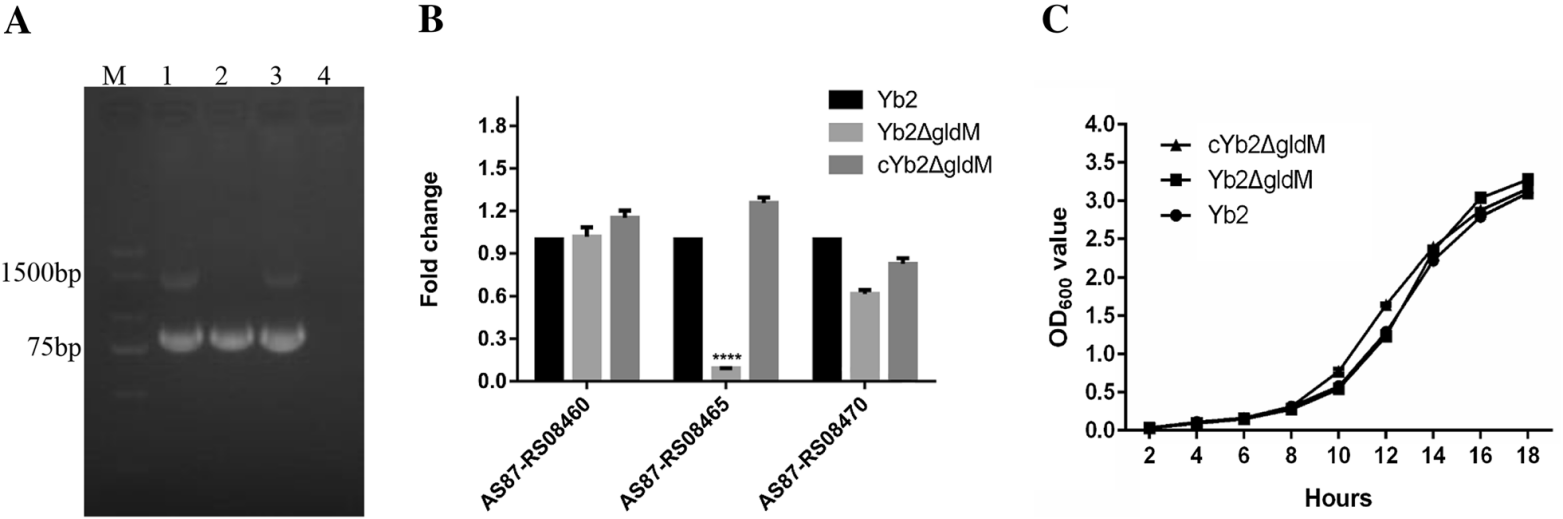
**Identification of the mutant strain Yb2Δ*gldM*** **and complementation strain cYb2Δ*gldM.*** **A** PCR amplification. M: DL2000 DNA Marker (Vazyme, Nanjing, China). Lane 1: the *R. anatipestifer gldM* gene and 16S rRNA were amplified from the wild-type strain Yb2 with the primer pairs AS87_RS08465-F/AS87_RS08465-R and RA16S rRNA-F/RA 16S rRNA-R, respectively, generating a 1560-bp fragment of *gldM* and a 792-bp fragment of *R. anatipestifer* 16S rRNA, respectively. Lane 2: a 792-bp fragment of RA 16S rRNA was amplified with the primer pair RA16S rRNA-F/RA 16S rRNA-R, but no 1560-bp fragment of the RA *gldM* gene was amplified with the primer pair AS87_RS08465-F/AS87_RS08465-R from the mutant strain Yb2Δ*gldM*. Lane 3: both the *R. anatipestifer gldM* gene and 16S rRNA were amplified from the complementation strain cYb2Δ*gldM* with the primer pairs AS87_RS08465-F/AS87_RS08465-R and RA16S rRNA-F/RA 16S rRNA-R, respectively, generating a 1560-bp fragment of *gldM* or a 792-bp fragment of RA 16S rRNA, respectively. Lane 4: distilled water was used as a non-strain control. **B** qPCR analyses of the polar effect in the mutant strain Yb2Δ*gldM*. No polar effect was observed. **C** Bacterial growth curves: no significant differences were detected among the *R. anatipestifer* wild-type strain Yb2, mutant strain Yb2Δ*gldM*, and complementation strain cYb2Δ*gldM*.

The mutant strain Yb2Δ*gldM*, in which the Tn*4351* transposon was inserted at nucleotide position 1307 bp of the *AS87_RS08465* gene, was identified in our previous study [25]. PCR amplification using the primers 16S rRNA-F/16S rRNA-R and RA_08465-F/RA_08465-R produced a 792-bp fragment of 16S rRNA and a 1560-bp fragment of *gldM*, respectively, in the wild-type strain Yb2 (lane 1) and complementation strain cYb2Δ*gldM* (lane 3), whereas no 1560-bp fragment was amplified from the mutant strain Yb2Δ*gldM* because of the insertion (Figure 1A). No polar effect of the *gldM* mutation was found by qPCR analyses on the adjacent genes (Figure 1B). The *AS87_RS08465* gene is 1560 bp in length and encodes a predicted 520-amino-acid GldM protein. A phylogenetic tree showed that *R. anatipestifer* and many other members of the phylum *Bacteroidetes* encode this protein [12]. GldM was originally identified as a protein required for bacterial gliding motility [22, 36], and a further study confirmed that GldM is a component of T9SS and involved in protein secretion [23, 37]. The growth rates of the wild-type strain Yb2, mutant strain Yb2*gldM*, and complementation strain cYb2*gldM* in TSB medium did not significantly differ (Figure 1C).

### Identification and localization of GldM

**Figure 2:**
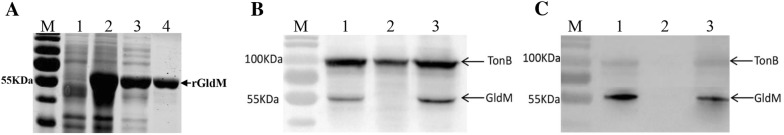
**Expression and identification of** ***R. anatipestifer*** **GldM. A** SDS-PAGE analysis of recombinant GldM (rGldM) expression. Lane M: PageRuler Prestained Protein Ladder (Thermo Scientific, Waltham, MA, USA); lane 1: *E. coli* BL21(DE3) transformed with pET-30a(+), with IPTG induction; lane 2: *E. coli* BL21(DE3) transformed with pET-*gldM*, with IPTG induction; lane 3: supernatant from *E. coli* BL21(DE3) transformed with pET-*gldM*, with IPTG induction; lane 4: purified rGldM. **B** Identification of the complementation strain cYb2*gldM* using a Western blotting assay. Lane 1: whole-cell proteins from the wild-type strain Yb2; lane 2: whole-cell proteins from the mutant strain Yb2Δ*gldM*; lane 3: whole-cell proteins from the complementation strain cYb2Δ*gldM.* **C** Subcellular localization of GldM in *R. anatipestifer* using a Western blotting assay. Lane 1: whole-cell proteins from the wild-type strain Yb2; lane 2: extract of cytoplasmic proteins from Yb2; lane 3: extract of membrane proteins from Yb2. A second antibody directed against the TonB-dependent receptor of *R. anatipestifer* was used to control for protein loading in both assays.

Histidine-tagged rGldM was efficiently expressed in *E. coli* BL21(DE3). As shown in Figure 2A, *E. coli* BL21(DE3) transformed with pET-30a(+) displayed no GldM (57-kDa) band (lane 1). The presence of a 57-kDa band was indicated in lanes 2 and 3 of a Coomassie blue-stained SDS-PAGE gel in which *E. coli* BL21(DE3) transformed with pET-*gldM* and its supernatant were loaded, respectively. After purification with HisTrap affinity columns, rGldM was identified as a single band in the gel (lane 4).

The expression of GldM in strains Yb2, Yb2Δ*gldM*, and cYb2Δ*gldM* was determined by Western blotting. As shown in Figure 2B, a 57-kDa band was detected in the wild-type strain Yb2 (lane 1) and complementation strain cYb2Δ*gldM* (lane 3) but was absent in the mutant strain Yb2Δ*gldM* (lane 2), indicating that GldM is encoded by the *AS87_RS08465* gene and that the expression of GldM is rescued in the complementation strain cYb2*gldM*. This analysis clearly indicated that the *AS87_RS08465* gene was successfully disrupted in the mutant strain Yb2Δ*gldM*.

To determine the subcellular location of GldM in *R. anatipestifer*, the cytoplasmic and membrane fractions of bacterial cells were extracted and analyzed by Western blotting. As shown in Figure 2C, a single 57-kDa band corresponding to GldM was detected in whole-cell proteins from the wild-type strain Yb2 (lane 1, positive control) and purified cytomembrane fraction (lane 3). No band was found in cytoplasmic proteins from Yb2 (lane 2), indicating that GldM is expressed in the cytomembrane of *R. anatipestifer*.

### *Riemerella anatipestifer* mutant strain Yb2Δ*gldM* is defective in gliding motility

**Figure 3:**
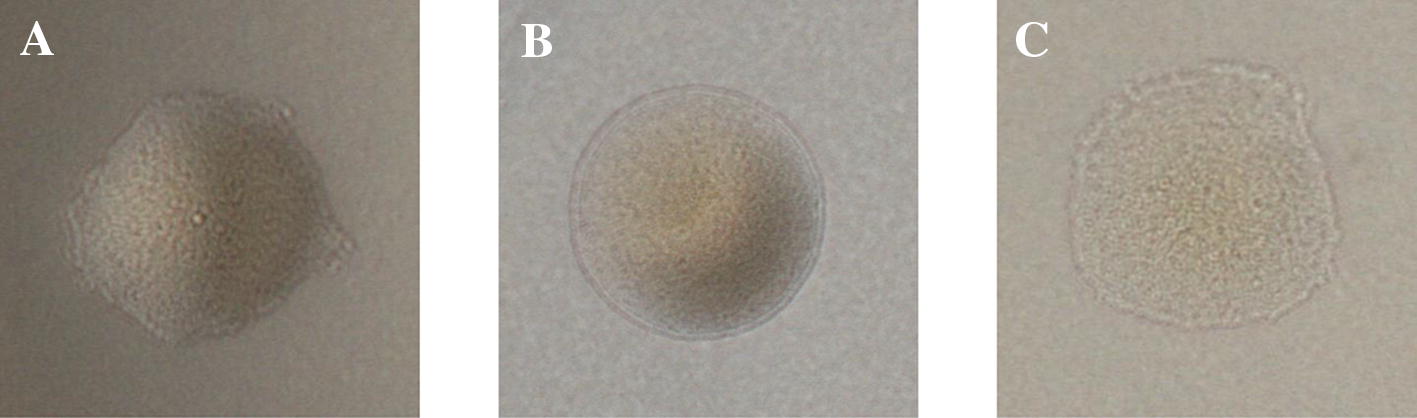
**Photomicrographs of** ***R. anatipestifer*** **colonies.** Colonies were grown for 24 h at 37 °C on TSA plates. Photomicrographs were taken with a Photometrics CoolSNAP_cf_^2^ camera mounted on a phase-contrast microscope. **A** Wild-type *R. anatipestifer* Yb2. **B** Mutant strain Yb2Δ*gldM*. **C** Complementation strain Yb2Δ*gldM*.

A transposon insertion in *gldM* resulted in the complete loss of *F. johnsoniae* motility, as reported previously [22]. In our study, the motility phenotypes of the wild-type strain Yb2, mutant strain Yb2Δ*gldM*, and complementation strain cYb2Δ*gldM* were examined. When cultured on 0.5% TSA plates, the wild-type strain Yb2 formed spreading colonies, whereas the mutant strain Yb2Δ*gldM* formed nonspreading colonies (Figures 3A and B). The gliding motility of the *R. anatipestifer* complementation strain cYb2Δ*gldM* on the agar surface was restored (Figure 3C).

### Mutant strain Yb2Δ*gldM* displays defective protein secretion

**Figure 4:**
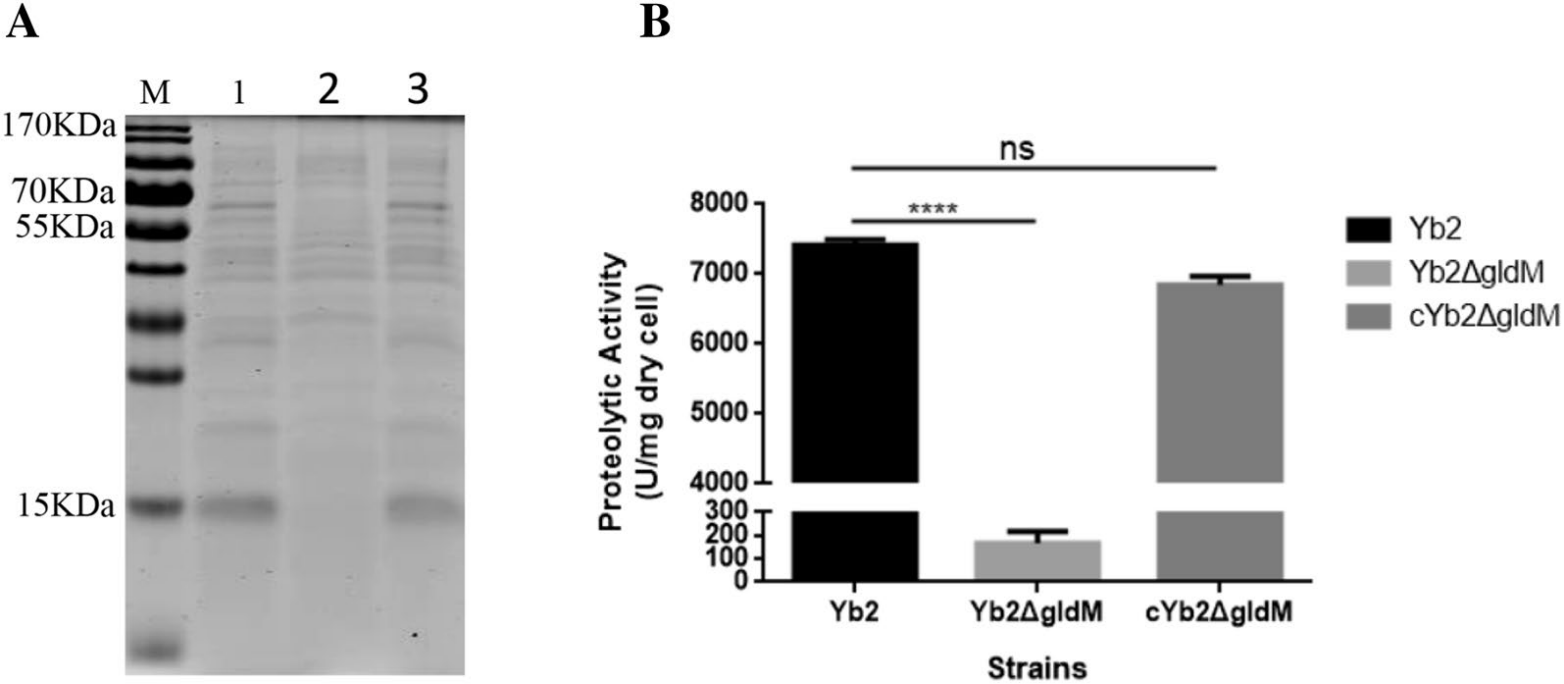
**Analysis of secreted proteins. A** SDS-PAGE profile of extracellular proteins. Lane 1: wild-type strain Yb2; lane 2: mutant strain Yb2∆*gldM*; lane 3: complementation strain cYb2∆*gldM*. Strains were incubated with shaking until cultures reached an OD_600_ of 0.8. The same volume of concentrated cell-free supernatant from each strain was separated by SDS-PAGE, and proteins were visualized with Coomassie Brilliant Blue staining. **B** Determination of proteolytic activity. The error bars represent the standard deviation calculated from three independent experiments performed in triplicate (****p < 0.0001; ns, *p *> 0.05)

T9SS is involved in the secretion of many proteins, such as the soluble extracellular chitinase ChiA, in the environmental bacterium *F. johnsoniae* [16, 38] and in the secretion of gingipain proteases and cell-surface adhesins in *Porphyromonas gingivalis*, which have been confirmed to be virulence factors in periodontitis [14, 23]. Cell-free culture fluids of the wild-type strain Yb2, mutant strain Yb2Δ*gldM* and complementation strain cYb2Δ*gldM* were collected and examined with SDS-PAGE to detect any differences in secreted proteins. We found fewer protein bands or smaller quantities of protein in the culture fluid from the mutant strain Yb2Δ*gldM* than from that of the wild-type strain Yb2 and complementation strain cYb2Δ*gldM* (Figure 4A).

The proteins in the cell-free culture fluids of strains Yb2, Yb2Δ*gldM* and cYb2Δ*gldM* were identified with LC–MS/MS. Our results indicate that the mutant strain Yb2Δ*gldM* was defective in the digestion of proteins (Figure 4B), consistent with the fact that the enzymes involved in the digestion of these polymers are secreted by T9SS. In addition, 165 proteins were differentially identified in the cell-free culture fluids of Yb2Δ*gldM* compared to those of Yb2 (Additional file 2). Among these proteins, nine had typical T9SS CTDs (Table 2) and could be predicted as T9SS proteins. The other proteins may have had novel targeting sequences or were released by an indirect process that involved T9SS. The complementation strain cYb2Δ*gldM* recovered most of the proteins secreted by the mutant strain Yb2Δ*gldM*, and only 45 proteins were differentially expressed between cYb2Δ*gldM* and Yb2 (Additional file 3). Moreover, the nine secreted proteins with the typical T9SS CTDs were all recovered in the complementation strain cYb2Δ*gldM* (Table 2).

**Table 2:** **Predicted** ***R. anatipestifer*** **Yb2 proteins secreted by T9SS identified with LC–MS/MS**

Locus tag	Molecular mass (KD^a^)	T9SS CTD^b^	Proteins	Predicted protein function	Relative change of protein^c^	Relative change of protein^d^
AS87_RS03090	13.49	+	Uncharacterized protein	Unknown	6.0762	1.6765
AS87_RS06600	117.29	+	Pkd domain containing protein	Collagenolytic of the catalytic	0.1004	1.1848
AS87_RS07295	93.945	+	Fibronectin type iii domain protein	Serine-type endopeptidase activity	0.0274	0.9055
AS87_RS02625	39.812	+	Endonuclease i	Endonuclease activity	0.2592	0.7272
AS87_RS00980	68.225	+	Metallophosphoesterase (MPPE)	Acid phosphatase activity	0.0434	1.3085
AS87_RS04190	77.52	+	Subtilisin-like serine protease	Serine-type endopeptidase activity	0.2041	1.1190
AS87_RS03200	117.12	+	Uncharacterized protein	Unknown	0.0063	1.2016
AS87_RS02020	83.131	+	Immunoreactive 84 kDa antigen pg93	Metal ion binding	0.3273	1.1742
AS87_RS00835	161.5	+	Peptidase s8 and s53 subtilisin kexinsedolisin	Serine-type endopeptidase activity	0.0045	1.2558

^a^Proteins in cell-free culture fluid from the wild-type strain Yb2, mutant strain Yb2Δ*gldM* and complementation strain cYb2Δ*gldM* were analyzed with LC–MS/MS.

^b^T9SS CTD identified with BLASTP analysis. + Indicates that the protein has a CTD.

^c^Relative difference in protein secretion between Yb2Δ*gldM* and Yb2.

^d^Relative difference in protein secretion between cYb2Δ*gldM* and Yb2.

### *gldM* disruption affects gene expression

Strand-specific Illumina RNA-Seq analysis was used to investigate the differentially expressed genes among the wild-type strain Yb2, mutant strain Yb2Δ*gldM* and complementation strain cYb2Δ*gldM*. In total, 15 genes were upregulated or downregulated in the mutant strain Yb2Δ*gldM* relative to their expression in the wild-type strain Yb2. qPCR confirmed that six genes in the mutant strain Yb2Δ*gldM* were upregulated > 2-fold at the transcription level (Table 3). The *AS87_RS00230* gene encodes the protein identified as SprT, which is a component of T9SS. The *AS87_RS09480* gene encodes a protein involved in “pilus twitching motility”. *AS87_RS08665* encodes a protein annotated as the Tat pathway signal sequence domain-containing protein. The *AS87_RS02915*, *AS87_RS09140*, and *AS87_RS02755* genes encode hypothetical proteins. Compared with the wild-type strain Yb2, five genes were downregulated > 2-fold in the mutant strain Yb2Δ*gldM*. The *AS87_RS01360* and *AS87_RS01350* genes, respectively, encode the hemolysin D protein and a CzcA family member, which is a heavy metal efflux pump. The putative product of the *AS87_RS01355* gene is a transporter. Both the *AS87_RS07850* and *AS87_RS01365* genes encode hypothetical proteins. The differentially expressed genes in the mutant strain Yb2Δ*gldM* were all restored in the complementation strain cYb2Δ*gldM* (Table 3). The gliding motility protein SprT is a component of T9SS and is involved in protein secretion and cell movement. The transporter encoded by *AS87_RS01355* is also involved in protein secretion. These results suggest that *AS87_RS08465* regulates genes that are mainly responsible for protein secretion and gliding motility in *R. anatipestifer.*

**Table 3:** **qPCR verification of differentially expressed genes in the mutant strain Yb2Δ** ***gldM*** **and complementation strain cYb2Δ** ***gldM***

Gene locus^a^	Description of genes	2^−ΔΔCt^	
		Yb2Δ*gldM*	cYb2Δ*gldM*
*AS87_RS00230* ^b^	PorT protein	3.5925	1.472
*AS87_RS02915*	Hypothetical protein	2.445	1.3755
*AS87_RS09140*	Hypothetical protein	2.0875	1.2058
*AS87_RS09480*	Twitching motility protein PilT	2.06	1.2397
*AS87_RS02755*	Hypothetical protein	2.0533	0.9432
*AS87_RS08665*	Tat pathway signal sequence domain-containing protein	2.03	1.3947
*AS87_RS09585*	Hypothetical protein	1.6533	1.1028
*AS87_RS08925*	SusC/RagA family TonB-liked outer membrane protein	1.4225	1.6531
*AS87_RS08050*	Carbohydrate-bonding protein	1.3875	0.8593
*AS87_RS04485*	Cbb3-type cytochrome oxidase component FixQ	1.35	1.021
*AS87_RS07185*	Hypothetical protein	1.28	1.0352
*AS87_RS09360*	Hypothetical protein	1.2725	1.1408
*AS87_RS05780*	Hypothetical protein	1.12	0.8526
*AS87_RS05060*	Hypothetical protein	1.0733	0.7474
*AS87_RS10475*	Hypothetical protein	1.0233	0.8745
*AS87_RS08045*	RagA family TonB-liked outer membrane protein	0.985	1.2256
*AS87_RS00555*	RNA polymerase sigma24 factor	0.98	1.0867
*AS87_RS08885*	Hypothetical protein	0.947	1.591
*AS87_RS09380*	Hypothetical protein	0.945	1.0497
*AS87_RS01535*	Hypothetical protein	0.93	1.2226
*AS87_RS05020*	Hypothetical protein	0.9175	0.895
*AS87_RS09995*	Hypothetical protein	0.8825	1.4948
*AS87_RS08585*	ABC transporter	0.8425	1.5157
*AS87_RS00880*	ABC transporter related protein	0.6175	0.9659
*AS87_RS08470* ^b^	Gliding motility protein GldN	0.615	0.7813
*AS87_RS07850*	Hypothetical protein	0.47	0.9075
*AS87_RS01360*	Hemolysin D	0.34	0.5586
*AS87_RS01365*	Hypothetical protein	0.33	0.8645
*AS87_RS01350*	Heavy metal efflux pump, CzcA family	0.295	0.7846
*AS87_RS01355*	Transporter	0.225	0.6925

^a^Based on the *R. anatipestifer* Yb2 genome (accession number: CP007204).

^b^These genes encode T9SS components.

### Deletion of *gldM* reduces bacterial adherence and invasion capacities

**Figure 5:**
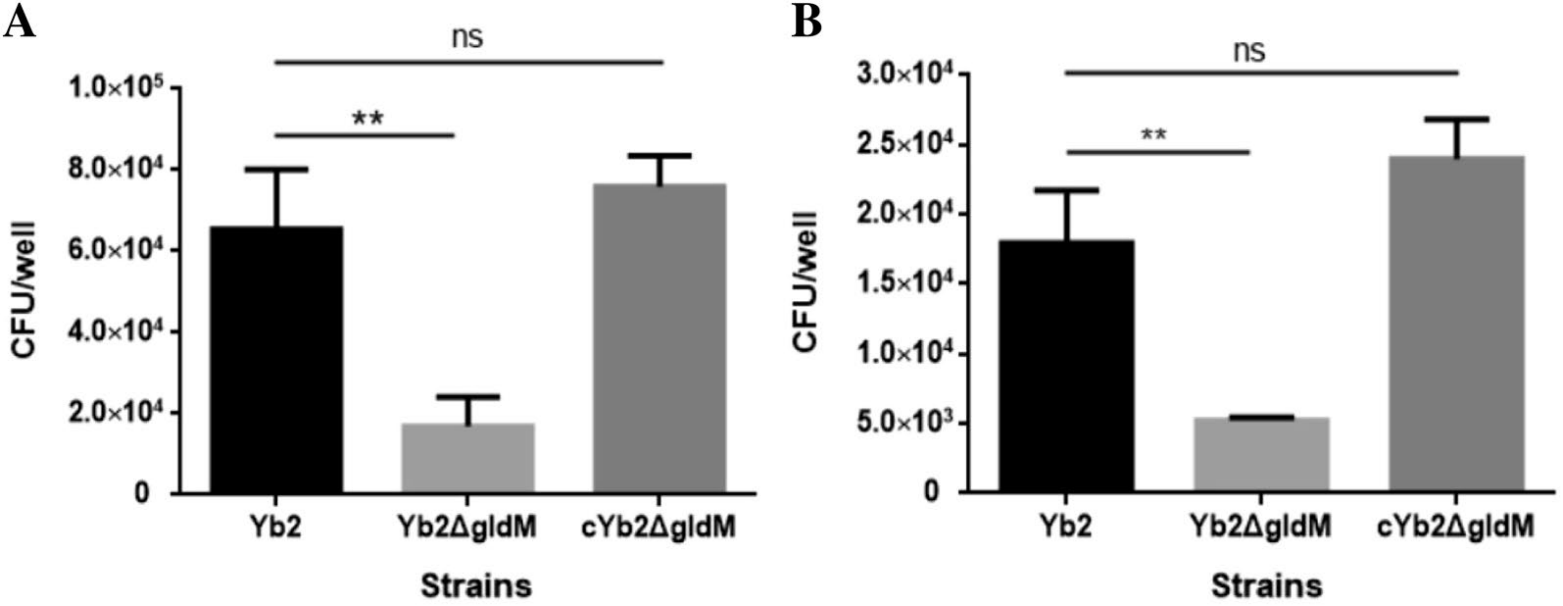
**Bacterial adherence (A) and invasion (B) assays.** The assays were performed on Vero cells, and the cells were infected with each strain at a MOI of 50. The data represent the counts of bacteria bound to or having invaded Vero cells in each well of a 24-well plate. The data are presented as the means ± standard deviations of three independent experiments (**p < 0.01; ns, *p *> 0.05).

The bacterial adherence and invasion capacities of the wild-type strain Yb2, mutant strain Yb2Δ*gldM*, and complementation strain cYb2Δ*gldM* were determined in Vero cells. When cells were infected with *R. anatipestifer* at a MOI of 50, the number of cell-adherent Yb2Δ*gldM* bacteria was 1.69 × 10^4^ CFU/well, which was significantly lower than the numbers of Yb2 (6.54 × 10^4^ CFU/well) and cYb2Δ*gldM* (7.59 × 10^4^ CFU/well) cell-adherent bacteria. When the cell-invasive bacteria were counted, the number of Yb2Δ*gldM* was 5.24 × 10^3^ CFU/well, almost threefold lower than the number of Yb2 (1.79 × 10^4^ CFU/well) or cYb2Δ*gldM* (2.40 × 10^4^ CFU/well). These results demonstrate that mutation of the *R. anatipestifer gldM* gene significantly reduces bacterial adherence and invasion (Figures 5A and B).

### Mutation of the *gldM* gene attenuates bacterial virulence

**Figure 6:**
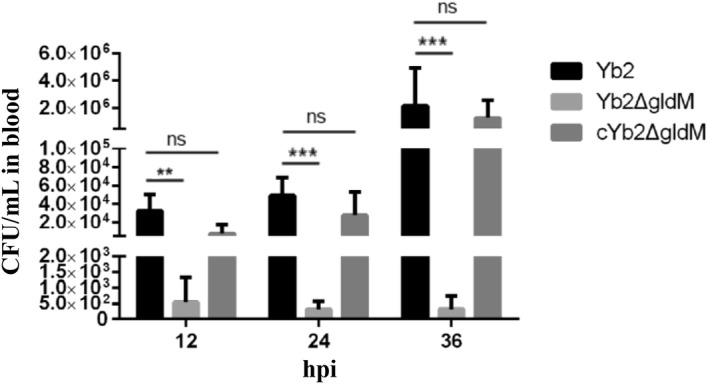
**Determination of bacterial loads.** Bacterial loads in the blood of ducks infected with *R. anatipestifer* Yb2, Yb2∆*gldM*, or cYb2∆*gldM* were determined at 12, 24, and 36 hpi. Bacterial colony-forming units were counted and analyzed. The data are presented as the means ± standard deviations of ten infected ducks and were analyzed using two-tailed independent Student’s *t* test. Asterisks indicate statistically significant differences between groups (**p < 0.01; ***p < 0.001; ns, *p *> 0.05).

In our previous study, the LD_50_ of the mutant strain Yb2Δ*gldM* was shown to be 1.97 × 10^7^ CFU, so its virulence was attenuated 184-fold compared with that of the wild-type strain Yb2 (1.07 × 10^5^ CFU) [25]. In this study, the bacterial loads in the blood of infected ducks were quantified to investigate the role of the *AS87_RS08465* gene in systemic infection in vivo. The bacterial loads of the mutant strain Yb2Δ*gldM* in infected duck’s blood were 5.45 × 10^2^ CFU/mL, 3.12 × 10^2^ CFU/mL, and 3.2 × 10^2^ CFU/mL at 12, 24, and 36 hpi, respectively, whereas the bacterial loads of the wild-type strain were 3.26 × 10^4^ CFU/mL, 4.9 × 10^4^ CFU/mL, and 2.18 × 10^6^ CFU/mL at 12, 24, and 36 hpi, respectively (Figure 6), indicating that deletion of the *gldM* gene significantly attenuated the virulence of *R*. *anatipestifer.* Bacterial recovery from the blood of infected ducks was similar after infection with the complementation strain cYb2Δ*gldM* and wild-type strain Yb2.

## Discussion

Genomic analyses have shown that T9SSs, also known as the “Por secretion system”, are common in members of the phylum *Bacteroidetes*, including *F. johnsoniae*, *P. gingivalis*, *Tannerella forsythia*, and *R*. *anatipestifer* [12]. This novel protein secretion system, which has only recently been recognized and studied, functions in bacterial gliding motility and protein secretion [23, 39, 40]. In *F. columnare*, proteases and chondroitin sulfate lyases, which are known virulence factors, are secreted via T9SS [41]. In *P. gingivalis*, Arg-gingipain, Lys-gingipain, and Skp protein, which are virulence factors that cause human tissue damage, are also secreted by T9SS [14, 42, 43].

GldM is a known cytoplasmic membrane component of the *F. johnsoniae* T9SS [22]. Therefore, GldM is potentially involved in harvesting cellular energy to power secretion and deliver motility adhesions [19]. Braun demonstrated that GldM is required for gliding in *F. johnsoniae* [22]. In the present study, Western blotting analysis showed that *R. anatipestifer* GldM was localized to the cytomembrane and that the mutant strain Yb2Δ*gldM* was defective in gliding motility, forming nonspreading colonies on the surface of agar. These results indicate that GldM is a membrane protein required for gliding in *R*. *anatipestifer*.

In *F. johnsoniae*, *gldK*, *gldL*, *gldM*, and *gldN* clustered together in the genome, and their products (GldK, GldL, GldM, and GldN, respectively) may form a complex [36]. In this study, qPCR revealed that the disruption of *gldM* dramatically downregulated the expression of *gldN*. The expression of the genes encoding a transporter (AS87_RS01355), heavy metal efflux pump (AS87_RS01350), hemolysin D (AS87_RS01360) and two hypothetical proteins (AS87_RS01365 and AS87_RS07850) was also downregulated in the *gldM* deletion mutant strain Yb2Δ*gldM*. These results suggest that *gldM* is associated with the expression of several genes that may work together in protein secretion. Several reports have demonstrated relationships between SprT and other T9SS components, and SprT is also required for the secretion of SprB, RemA, and chitinase, which function like GldM in *F. johnsoniae*, where *gldK*, *gldM*, *gldN* and *porT* are components of T9SS and are associated with bacterial gliding motility [19]. Our results also showed that the expression of *sprT* (an orthologue of *port* in *P. gingivalis*) was significantly elevated in the mutant strain Yb2Δ*gldM*. In addition, our LC–MS results showed that many secretion proteins were present at higher levels in the cell-free culture of Yb2Δ*gldM* than in Yb2, which may be related to the upregulation of the *sprT* gene, and the change in SprT secretion may compensate for the *gldM* deletion. Future studies are required to clarify the relationships of these proteins in various processes.

The *sprT* gene encodes the T9SS component SprT, which is involved in the protein secretion and virulence of *R*. *anatipestifer* [21]. However, the functions of other T9SS components in *R*. *anatipestifer* are still unclear. In this study, deletion of the *R*. *anatipestifer gldM* gene, which encodes a component of T9SS, significantly reduced the bacterial adherence and invasion capacities and the bacterial loads in infected duck blood compared with those of the wild-type strain Yb2. These findings provide powerful evidence that T9SS plays an important role in the pathogenicity of *R*. *anatipestifer.*

The SDS-PAGE profiles of cell-free cultures revealed that the levels of many secreted proteins were reduced or that these proteins were absent when *gldM* was mutated. LC–MS/MS demonstrated that more than 100 proteins were differentially secreted from the mutant strain Yb2Δ*gldM* compared to the wild-type strain Yb2. Genomic analysis showed that nine of the differentially expressed proteins had CTDs that were predicted to target them for secretion by T9SS. All of the CTDs belong to the TIGRFAM protein domain family TIGR04183 (and are referred to as “type A CTDs”) [41], a conserved carboxyterminal domain of the extracellular proteinase family.

In summary, we showed that the *R*. *anatipestifer AS87_RS08465* gene encodes GldM, which is required for gliding motility, protein secretion, and bacterial virulence of the bacterium. More than 100 proteins were differentially secreted from the mutant strain Yb2Δ*gldM*, suggesting their importance in the full virulence of *R*. *anatipestifer*. The molecular mechanisms by which these proteins act in bacterial virulence require further clarification, which should facilitate the development of effective strategies to control *R. anatipestifer* infection.

## Additional files

**Additional file 1.** **Primers used for real-time PCR analysis.**

**Additional file 2.** **Differentially secreted proteins of the wild-type strain Yb2 and mutant strain Yb2Δ*gldM***.

**Additional file 3.** **Differentially secreted proteins of the wild-type strain Yb2 and complementation strain cYb2Δ*gldM***.

## Abbreviations

T9SS: type IX secretion system
CTDs: C-terminal domains
TSB: tryptic soy broth medium
TSA: tryptic soy agar
ELISA: enzyme-linked immunosorbent assay
PCR: polymerase chain reaction
CFU: colony forming unit
SDS-PAGE: sodium dodecyl sulfate polyacrylamide gel electrophoresis
LD_50_: median lethal doses
