# Supplementary material for: Riemerella anatipestifer GldM is required for bacterial gliding motility, protein secretion, and virulence
Source: Vet Res. 2019 Jun 4;50:43. doi: 10.1186/s13567-019-0660-0 (PMC6549377; doi:10.1186/s13567-019-0660-0)
Supplement: Supplementary file 1 — Additional file 1. Primers used for real-time PCR analysis. [file 13567_2019_660_MOESM1_ESM.docx]

| **Gene tag^a^** | **Forward primer sequences (5’ - 3’)** | **Reverse primer sequences (5’ - 3’)** | **Products**  **(bp)** |
| --- | --- | --- | --- |
| AS87_RS00230 | CGTGGGCGTTATCATCTTCA | ATGCCAAGGCTCCATTCACT | 201 |
| AS87_RS02915 | GGTGGAAAAGCAGGATACGC | TTGAACTGCCCATTTGTTGG | 156 |
| *AS87_RS09140* | GCTGCCGTACATGTTTGGATT | TGAAGGCTAAACGCGTCAATTA | 152 |
| *AS87_RS09480* | TTTAAAAATGGAAGCCAGAAAAGC | TTGCGGTTAAGACAGGCAAA | 218 |
| *AS87_RS02755* | AAATGCTGATATTTTCTTTCCTTTTGA | TTTTCGGAGTTAAGATTGCCAAA | 195 |
| *AS87_RS08665* | ACAAAGGAGAAGGGGCTTACATT | GGCAGCTCTGTTTCCAGGAG | 194 |
| *AS87_RS09585*  *AS87_RS08925*  *AS87_RS08050* | AAAAATAGCGTTCGTTTTGAGGTC  ATGCTACGGCGGCAGTTTAT  CAGAAGCACAGGCGATGCTA | TCGAAAAATAACTTTGTACTGAATGGA  CTGCAAGTCCTGCATTTTGG  AATCTTGCCAACGCAAACCT | 218  201  198 |
| *AS87_RS04485* | GGCTTTTACCAAAGCCTAGCAC | AAGGAGCATTAGCTTCGTCTTGA | 112 |
| *AS87_RS07185*  *AS87_RS09360*  *AS87_RS05780* | GAAGCAAGGCTAAATCCGCTAA  TCCTTCAATGAACATTGCTGAAGT  CAAACAAAGAGTAGCAGAAGATGGA | ACGCTCCTCCCAAATCACTC  TCACAATAAATCCAACCGTTATTCTC  CAGAACCTGCCATTTCAGTCC | 229  150  182 |
| *AS87_RS05060* | TCTTTCCTTGAGCGAATATTTTGA | TTCATCGCCAAGAATTCCAA | 232 |
| *AS87_RS10475* | TGGGCAGTCGTAAATATTGGAA | TCGCTAAGAATACAAGTGGACCAA | 100 |
| *AS87_RS08045* | GCGGCATCTTTCAACATCAA | GGGTCCCAATTAGCAAACCA | 203 |
| *AS87_RS00555* | AACAACCGACTGGCACAAAA | CGTACGATGCTAATGGCTTCA | 215 |
| *AS87_RS08885* | GGCAAAAGCCTCAAAAGCAT | GAAAACGCACCTCCTTTTCG | 171 |
| *AS87_RS09380*  *AS87_RS01535*  *AS87_RS05020*  *AS87_RS09995*  *AS87_RS08585*  *AS87_RS00880*  *AS87_RS07850*  *AS87_RS01360*  *AS87_RS01365*  *AS87_RS08470*  *AS87_RS01350*  *AS87_RS01355* | TCCTTCAATGAACATTGCTGAAGT  TCCGCAAAAATGGGAATTAGA  TGGACTTGCTACTTCGGGCAAAAGTACTCTCAAATTGGGCTGA  TATTGGTCCAAACGGCTGTG  ATGAACCTACGGCAGGGCTA  AGGAGCTCAAGCCTCTGCTG  GGCGATGTTTTGGCGATATT  TGGGTTAGTGCGATTTGTGG  CCACAAACTATGGGGCAACA  CGAAGTAGCTGGTGCAGTGG  CGGTGGTAGTTGGGGTGTCT | TCACAATAAATCCAACCGTTATTCTC  CCGTTATATTCAGTTCCGTCAATTT  ACGATGAAATGGCGGCTTTA  TTGCGTGATACCACCTACCG  TCCCTATCTGAGGCATGTAGCC  CAGCATTTCTTCCCCTTGCT  TGTTCCCCCAGAAACGGTAG  GGCGATGTTTTGGCGATATT  TGCTCCCAAAAAGACAGCAG  CAACTGAGCCTCTGCATCGT  AAAGCCCCTTCCATGAGGTT  CCCTTCATATAATTGGCGATTAGC | 150  150  158  195  152  197  190  157  210  249  195  248 |

**Additional file 1 Primers used for real-time PCR analysis.**

^a^ Based on *R. anatipestifer* Yb2 genome (accession number: CP007204).
