# Supplementary material for: Riemerella anatipestifer GldM is required for bacterial gliding motility, protein secretion, and virulence
Source: Vet Res. 2019 Jun 4;50:43. doi: 10.1186/s13567-019-0660-0 (PMC6549377; doi:10.1186/s13567-019-0660-0)
Supplement: Supplementary file 2 — Additional file 2. Differentially secreted proteins of the wild-type strain Yb2 and mutant strain Yb2ΔgldM. [file 13567_2019_660_MOESM2_ESM.docx]

**Additional file 2 The differential secretory proteins of wild-type strain Yb2 and mutant strain Yb2Δ*gldM*.**

| **Locus**  **tag** | **Predicted**  **protein** | **Peptides^1^** | **Unique peptides^2^** | **Sequence coverage [%]** | **MW^3^ [kDa]** | **LFQ^4^ intensity**  **of Yb2(A)** | **LFQ intensity**  **of Yb2*ΔgldM* (B)** | **B/A** | |  |
| --- | --- | --- | --- | --- | --- | --- | --- | --- | --- | --- |
|  |  |  |  |  |  | **Mean** | **Mean** | **Ratio** | ***P* value** |  |
| AS87_RS00275 | Peptidylprolyl isomerase | 17 | 17 | 57.2 | 39.433 | 331220000 | 815183333.3 | 2.4612 | 0.048247 |  |
| AS87_RS02325  AS87_RS02975  AS87_RS03880  AS87_RS08385  AS87_RS04395  AS87_RS02880  AS87_RS00175  AS87_RS07270  AS87_RS02920  AS87_RS03090  AS87_RS01935  AS87_RS00415  AS87_RS00180  AS87_RS09225  AS87_RS03960  AS87_RS02285  AS87_RS02370  AS87_RS03290  AS87_RS09905  AS87_RS01705  AS87_RS08080  AS87_RS07620  AS87_RS05425  AS87_RS04950  AS87_RS03560  AS87_RS06305  AS87_RS06310  AS87_RS10015  AS87_RS02040  AS87_RS07595  AS87_RS04355  AS87_RS07795  AS87_RS05565  AS87_RS08185  AS87_RS02195  AS87_RS07800  AS87_RS08100  AS87_RS00770  AS87_RS01745  AS87_RS04050  AS87_RS06395  AS87_RS07175  AS87_RS07185  AS87_RS07245  AS87_RS07350  AS87_RS06300  AS87_RS08065  AS87_RS01225  AS87_RS01905 | Uncharacterized proteinSuccinate--CoA ligase [ADP-forming] subunit alphaNucleotide sugar dehydrogenaseFlavin reductase domain protein fmn-binding proteinElongation factor TuUncharacterized proteinAnkyinTetratricopeptide tpr_1 repeat-containing proteinTrigger factorUncharacterized proteinDihydrolipoyllysine-residue succinyltransferase component of 2-oxoglutarate dehydrogenase complexUncharacterized proteinCatalaseAconitase50S ribosomal protein L31 type BUncharacterized proteinUncharacterized proteinLong-chain fatty acid transport proteinUncharacterized proteinUncharacterized protein3,4-dihydroxy-2-butanone 4-phosphate synthaseEnolaseAmino acid/amide ABC transporter substrate-bindingprotein, haat familyUncharacterized proteinUncharacterized protein5-hydroxyisourate hydrolaseThioredoxin domain-containing proteinUncharacterized proteinOuter membrane receptor protein, mostly Fe transportDNA-directed RNA polymerase subunit alphaThrombospondin type 3 repeat-containing proteinUncharacterized proteinTonb-dependent receptorRhodanese domain proteinChaperone protein DnaKElongation factor TsSuccinate--CoA ligase [ADP-forming] subunit betaAminomethyltransferase60 kDa chaperoninUncharacterized proteinUncharacterized proteinProtein-disulfide reductaseUncharacterized proteinOuter membrane transport energization protein exbdUncharacterized proteinUncharacterized proteinPasta domain containing proteinUncharacterized proteinUncharacterized protein | 12  20  8  9  16  4  9  28  22  4  13  2  34  35  5  12  6  6  6  8  9  15  16  10  15  9  14  16  10  12  12  11  17  5  26  22  17  15  30  34  13  10  5  9  12  7  9  5  4 | 12  20  8  9  16  4  9  27  22  4  13  2  34  35  5  12  6  6  6  5  9  15  16  10  15  8  14  16  10  12  12  11  17  5  26  22  16  15  30  34  13  10  5  9  121  7  9  5  4 | 72.8  50.5  21.1  44.7  52.4  27.1  62  57.8  56.5  37.3  40.5  14  69.5  54.7  64.3  68.5  34.7  35.8  56.5  66.1  30.6  43  33.6  34.6  75.6  72.6  74.7  52.2  13.9  45.6  27.9  60.8  18.8  62.8  50.2  75.8  53.5  49  72.1  40.5  21.4  14.6  31.7  47.9  14  63.1  33.7  47.4  32.8 | 26.842  45.956  47.133  30.414  40.288  17.073  20.259  60.678  50.991  13.49  44.305  14.657  58.165  82.206  9.8212  16.918  26.746  21.082  13.293  19.518  41.83  46.494  70.227  32.803  13.454  15.478  20.638  39.924  96.961  37.132  53.52  21.656  90.142  10.478  68.236  35.041  42.85  39.77  57.336  106.92  78.701  76.892  19.093  21.54  101.68  16.552  38.68  13.741  14.529 | 143833333.3  16666666.67  12746500  17993000  506383333.3  7441000  871870000  579846666.7  52340000  242157333.3  2653480  4873600  1488500000  235853333.3  2548250  19577633.33  104001000  6679600  27495500  233693333.3  12077300  40895333.33  10714766.67  3710233.333  15442333333  496933333.3  608846333.3  95606000  499143333.3  13433600  94867333.33  35067666.67  416243333.3  18483200  93555333.33  229374800  77335500  10992666.67  125093000  636006666.7  37247066.67  86774000  14683600  17442033.33  617420000  168695000  20429000  11262533.33  14035666.67 | 569630000  80989666.67  73980000  52388000  1842033333  49187333.33  5966600000  2286466667  297730000  1471400000  141900000  10918733.33  3097466667  664006666.7  131180000  181096666.7  235283333.3  24262333.33  236846666.7  961113333.3  46965500  88780000  82444666.67  46083666.67  51986666667  1210663333  4907466667  262986666.7  2986533333  49787000  252673333.3  243380000  2351733333  144906666.7  670916666.7  2442100000  435740000  53758666.67  386190000  1497166667  587433333.3  385090000  43418666.67  219739000  1783033333  667286666.7  160306666.7  38634333.33  32070000 | 3.9603  17.0605  5.8039  2.9116  3.6376  6.6103  6.8435  3.9432  5.6884  6.0762  53.4769  2.2404  2.0809  2.8153  51.4785  9.2502  2.2623  3.6323  8.6140  4.1127  3.8887  2.1709  7.6945  12.4207  3.3665  2.4363  8.06033  2.7507  5.9833  3.7062  2.6634  6.9403  5.6499  7.8399  7.1713  10.6468  5.6344  4.8904  3.0872  2.3540  15.7713  4.4379  2.9570  12.5982  2.8879  3.9556  7.8470  3.4303  2.2849 | 0.000486  0.00141  0.001614  0.001896  0.002735  0.003029  0.003859  0.004328  0.005369  0.006052  0.00693  0.007319  0.007583  0.007796  0.008139  0.009563  0.011514  0.011751  0.01208  0.012548  0.01455  0.015193  0.015316  0.016631  0.016737  0.016944  0.016949  0.017674  0.019721  0.022354  0.022645  0.02292  0.024031  0.026153  0.027708  0.029991  0.032395  0.03379  0.033959  0.03398  0.034651  0.035626  0.036743  0.039186  0.040169  0.045336  0.047144  0.047784  0.048091 |  |
| AS87_RS08210  AS87_RS06525  AS87_RS05440  AS87_RS00035  AS87_RS10295  AS87_RS06600  AS87_RS09010  AS87_RS05225  AS87_RS06510 | Uncharacterized proteinRagb/susd domain proteinRagb/susd domain proteinUncharacterized proteinUncharacterized proteinPkd domain containing proteinUncharacterized proteinGlucokinaseUncharacterized protein | 6  29  24  5  3  65  5  8  11  34  16  14  21  11  27  15  13  7  19  22  15  43  46  8  18  21  9  1  5  44  17  25  34  10 | 6  29  24  5  3  65  5  8  11  33  16  14  21  11  27  15  13  7  19  21  15  43  46  8  18  21  9  1  5  44  17  22  34  10 | 31.6  62.4  59.8  2.8  22.6  66  40.6  28.7  28.7  58.7  62.8  79.7  28.4  56.9  60.4  28.4  22.7  51.5  40.3  46.1.  42.5  48.8  41.8  25.1  22.4  35.6  33.2  5.5  16.4  30.1  13.9  26.9  30.3  23.2 | 27.513  54.642  54.229  175.46  20.916  117.29  23.059  34.549  46.14  93.945  39.812  22.828  102.41  22.384  58.972  65.656  71.841  21.426  68.225  77.52  51.224  117.12  153.79  39.232  90.777  83.131  32.681  23.392  37.31  167.27  137.67  109.12  161.5  74.333 | 212093333.3  2358466667  1881933333  71687666.67  43448333.33  38244333333  3330166667  49348666.67  1538866667  9865366667  1405253333  14375666667  50035666.67  3251866667  841420000  118942000  127649666.7  97546666.67  454376666.7  2711533333  128473333.3  3132500000  1308896667  23118000  109510000  117862000  84201000  4165966.667  11308966.67  340050000  71359333.33  161946666.7  311486666.7  333023333.3 | 42668000  469440000  602606666.7  21155000  21698000  3840466667  230306666.7  17615666.67  100557666.7  270640000  364223333.3  6740133333  16595333.33  135303333.3  457770000  45420666.67  44248333.33  23721000  19740650  553583333.3  35867666.67  19736333.33  123283000  11368933.33  37669000  38583000  36156333.33  1969000  4199433.333  60854000  18225966.67  73381666.67  1410450  146644333.3 | 0.2012  0.1990  0.3202  0.2951  0.4994  0.1004  0.0692  0.3570  0.0653 | 0.000116  0.000804  0.000993  0.001329  0.001495  0.002718  0.004908  0.005153  0.007275 |  |
| AS87_RS07295  AS87_RS02625  AS87_RS03125  AS87_RS01940  AS87_RS06505  AS87_RS06495  AS87_RS03600  AS87_RS06270  AS87_RS01300  AS87_RS00980  AS87_RS04190  AS87_RS02330  AS87_RS03200  AS87_RS07755  AS87_RS00245  AS87_RS01805  AS87_RS02020  AS87_RS00105  AS87_RS04235  AS87_RS02300  AS87_RS02200  AS87_RS01130  AS87_RS06530  AS87_RS00835  AS87_RS04340 | Fibronectin type iii domain protein  Endonuclease i  Uncharacterized protein  2-oxoglutarate dehydrogenase, e1 subunit  Uncharacterized protein  Uncharacterized protein Xaa-pro aminopeptidaseZn-dependent aminopeptidase Uncharacterized protein  Metallophosphoesterase (MPPE)  Subtilisin-like serine protease  Peptidase m20  Uncharacterized protein  Uncharacterized protein  Beta-ketoacyl-acyl-carrier-protein synthase iii  Transketolase domain-containing protein  Immunoreactive 84 kDa antigen pg93  4-hydroxy-tetrahydrodipicolinate synthase  Uncharacterized protein  Geranylgeranyl pyrophosphate synthase Yd repeat proteinPhosphoribosylformylglycinamidine synthaseTonb-dependent receptor plugPeptidase s8 and s53 subtilisin kexinsedolisin Uncharacterized protein |  |  |  |  |  |  | 0.0274  0.2592  0.4689  0.3317  0.0416  0.544045  0.3819  0.3466  0.2432  0.0434  0.2042  0.2792  0.0063  0.0942  0.4918  0.3440  0.3274  0.2122  0.4727  0.3713  0.1790  0.2554  0.4531  0.0045  0.4403 | 0.007732  0.012054  0.012411  0.013019  0.013377  0.012666  0.014445  0.014801  0.018917  0.020366  0.024286  0.026369  0.026632  0.026896  0.027477  0.034873  0.034895  0.049931  0.03843  0.038975  0.044918  0.045696  0.048029  0.048811  0.049277 |  |
| AS87_RS01525AS87_RS02115  AS87_RS02905AS87_RS06650  AS87_RS05935AS87_RS03155  AS87_RS07915AS87_RS07910  AS87_RS07500AS87_RS07155  AS87_RS06835  AS87_RS06825  AS87_RS04445AS87_RS08555  AS87_RS02580 | 3-oxoacid CoA-transferase, b subunit  Aspartate carbamoyltransferase  Tonb-dependent receptor plug  Uncharacterized protein  Oligopeptidase b  N5-carboxyaminoimidazole ribonucleotide synthase  Uncharacterized protein  Beta-lactamase  30S ribosomal protein S3  Yd repeat protein  Uncharacterized protein  Surface antigen (D15)  Uncharacterized protein  Pirin domain protein  Alkylhydroperoxide reductase/ thiol  specific antioxidant/ mal allergen | 8  3  2  3  3  4  2  3  2  96  6  3  7  4  1 | 1  3  2  3  3  4  2  3  2  96  6  3  7  4  1 | 12.5  10.4  6.8  4.7  4.5  10  7.1  8.3  7.4  51.7  12.5  3.6  23  13.7  5.4 | 49.493  33.753  89.571  62.97  79.307  41.416  34.305  41.411  25.937  260.25  69.034  89.071  40.353  32.397  105.47 | 4796150  2924250  14100500  6247700  67164500  42464500  6165200  12735850  123620660  5251333330  17604335  4113000  77199433  50484466  17386333 | 0  0  0  0  0  0  0  0  0  0  0  0  0  0  0 | -∞  -∞  -∞  -∞  -∞  -∞  -∞  -∞  -∞  -∞  -∞  -∞  -∞  -∞  -∞ | N  N  N  N  N  N  N  N  N  N  N  N  N  N  N |  |
| AS87_RS08155  AS87_RS01535  AS87_RS02925  AS87_RS06385  AS87_RS06070  AS87_RS06050  AS87_RS05980  AS87_RS00580  AS87_RS00655  AS87_RS01335  AS87_RS03530  AS87_RS07810  AS87_RS06985  AS87_RS06875  AS87_RS05400  AS87_RS05180  AS87_RS04840  AS87_RS04535  AS87_RS04955  AS87_RS08260  AS87_RS08335  AS87_RS08695  AS87_RS09640  AS87_RS09945  AS87_RS10205  AS87_RS00220  AS87_RS01460  AS87_RS01560  AS87_RS01850  AS87_RS02230  AS87_RS03925  AS87_RS06705  AS87_RS06605  AS87_RS06850  AS87_RS06325  AS87_RS00260  AS87_RS00270  AS87_RS00315  AS87_RS00500  AS87_RS00745  AS87_RS01085  AS87_RS01295  AS87_RS04125  AS87_RS07600  AS87_RS07495  AS87_RS07480  AS87_RS07335  AS87_RS07285  AS87_RS05415  AS87_RS05405  AS87_RS05385  AS87_RS05315  AS87_RS04810  AS87_RS04565  AS87_RS04525  AS87_RS04425  AS87_RS08255  AS87_RS09100  AS87_RS09790  AS87_RS10075  AS87_RS10155  AS87_RS10310  AS87_RS07505  AS87_RS05690  AS87_RS00800 | Uncharacterized protein  Uncharacterized protein  Probable endolytic peptidoglycan  transglycosylaseRlpA  Filamentation induced by camp protein fic  Uncharacterized protein  Peptidoglycan glycosyltransferase  Uncharacterized protein  Uncharacterized protein  Nlp/p60 protein  Uncharacterized protein  Sh3 type 3 domain protein  Alkyl hydroperoxide reductase/ thiol  specific antioxidant/ mal allergen  50S ribosomal protein L32  ATP-binding protein (Contains p-loop)  Rhodanese domain protein  Nlp/p60 protein  Enoyl-CoA hydratase/isomerase  30S ribosomal protein S9  RNA polymerase, sigma 54 subunit, rpon/sigl  Uncharacterized protein  Aminotransferase class-iii  Transcriptional regulator, padr family  ATP-dependent Clp protease proteolytic subunit  Uncharacterized protein  Erf family protein  Uncharacterized protein  Uncharacterized protein  Peptidase m14 carboxypeptidase a  Acyl-CoA dehydrogenase domain-containing protein  Pyridoxal-5'-phosphate-dependent protein  beta subunit  Bifunctional protein FolD  dTDP-4-dehydrorhamnose reductase  Uncharacterized protein  Dnak suppressor protein, putative  Iron-regulated ABC transporter membrane component sufb  Ribosome-binding ATPase YchF  Enoyl-[acyl-carrier-protein] reductase [NADH]  Uncharacterized protein  Thioredoxin domain-containing protein  Uncharacterized protein  Anthranilate synthase, component ii  Bifunctional purine biosynthesis protein PurH  Nitrogen-fixing nifu domain protein  50S ribosomal protein L17  50S ribosomal protein L22  50S ribosomal protein L23  Outer membrane transport energization protein exbd  Pur-alpha/beta/gamma DNA/RNA-binding protein  Thioredoxin  Uncharacterized protein  Uncharacterized protein  Transcription antitermination protein NusB  Transcription antitermination protein NusB  Transcription termination factor Rho  Fes assembly suf system protein  Ribosome hibernation protein yhbh  Phage shock protein a, pspa  Uncharacterized protein  Electron transfer flavoprotein alpha subunit  DNA methylase  Uncharacterized protein  Thioesterase superfamily protein  50S ribosomal protein L16  Ribose-phosphate pyrophosphokinase  Arsenate reductase and related protein | 2  1  2  6  2  3  5  1  3  3  3  8  2  5  1  1  5  2  4  1  1  4  3  1  4  2  2  2  6  7  5  8  2  5  9  5  6  7  1  3  3  12  5  5  5  3  3  7  3  6  5  3  4  2  3  4  9  4  3  6  1  2  3  8  3 | 2  1  2  6  2  3  5  1  3  3  3  8  2  5  1  1  5  2  4  1  1  4  3  1  4  2  2  2  6  7  5  8  2  5  9  5  6  7  1  3  3  12  5  5  5  3  3  7  3  6  5  3  4  2  3  4  9  4  3  6  1  2  3  8  3 | 13.4  6.6  18.3  16.2  14.6  3.8  15.1  5.6  23.1  26.2  9.4  67.1  22.4  38.7  9.5  4.6  21.7  16.4  8.4  6.8  3.6  38.5  23.2  10.5  20.5  12.2  16.5  7.1  15.7  20.3  20.4  34.8  15.6  46  21.4  13.5  28.7  38.3  10.8  39.4  19.5  28.4  55.1  30.5  35.8  32.3  25.8  66.1  38.4  22.3  35.4  20  11.6  3.5  40.7  44.8  40.8  11  13.4  22.8  20.8  17.2  26.4  32.7  23 | 20.8  23.04  14.145  43.14  18.539  89.5  42.66  22.109  18.75  14.23  30.151  16.909  8.028  15.64  14.929  26.764  27.524  14.425  56.354  15.714  43.416  12.627  25.048  16.615  21.383  21.707  12.286  42.59  41.181  38.305  32.434  32.72  19.435  14.482  53.948  40.697  29.997  23.95  11.779  11.572  21.203  56.055  8.6819  18.687  15.231  10.608  14.697  13.475  11.117  30.799  16.748  20.526  35.885  63.69  12.259  10.993  26.089  34.775  32.804  27.492  5.9387  14.493  13.87  34.229  11.863 | 8865850  0  0  0  0  0  0  0  0  0  0  0  0  0  0  0  0  0  0  0  0  0  0  0  0  0  0  0  0  0  0  0  0  0  0  0  0  0  0  0  0  0  0  0  0  0  0  0  0  0  0  0  0  0  0  0  0  0  0  0  0  0  0  0 | 0  2499300  968000  2975800  10386500  14534500  6958500  14335500  1760450  9239500  2489900  6750500  2767850  5376000  3046350  439055  9201400  3247850  4530600  4590100  2477550  1494450  3889150  3204400  5614500  4041600  14582333.3  8098333.33  15918766.7  16741000  8824133.33  22911333.3  20511333.3  14271666.7  14486666.7  43434333.3  6405966.67  25623333.3  28296666.7  5930966.67  6513666.67  28354666.7  18899333.3  13865666.7  10014866.7  6706766.67  45989333.3  25542666.7  21383200  12064300  39501333.3  13388766.7  3825533.33  3841533.33  19308666.7  61320333.3  60284000  14499600  11097200  39624333.3  5688733.33  4118200  7977133.33  53656666.7  16029333.3 | -∞  +∞  +∞  +∞  +∞  +∞  +∞  +∞  +∞  +∞  +∞  +∞  +∞  +∞  +∞  +∞  +∞  +∞  +∞  +∞  +∞  +∞  +∞  +∞  +∞  +∞  +∞  +∞  +∞  +∞  +∞  +∞  +∞  +∞  +∞  +∞  +∞  +∞  +∞  +∞  +∞  +∞  +∞  +∞  +∞  +∞  +∞  +∞  +∞  +∞  +∞  +∞  +∞  +∞  +∞  +∞  +∞  +∞  +∞  +∞  +∞  +∞  +∞  +∞  +∞ | N  N  N  N  N  N  N  N  N  N  N  N  N  N  N  N  N  N  N  N  N  N  N  N  N  N  N  N  N  N  N  N  N  N  N  N  N  N  N  N  N  N  N  N  N  N  N  N  N  N  N  N  N  N  N  N  N  N  N  N  N  N  N  N |  |
| AS87_RS01475 | Uncharacterized protein | 2 | 2 | 22.1 | 12.268 | 0 | 6996550 | +∞ | N |  |

^1^Peptide counts of the protein identified by LC-MS

^2^The specific peptide counts of the protein identified by LC-MS

^3^Mol. Weight of the protein

^4^Using the Label-free quantification method to quantify protein
