# Supplementary material for: Riemerella anatipestifer GldM is required for bacterial gliding motility, protein secretion, and virulence
Source: Vet Res. 2019 Jun 4;50:43. doi: 10.1186/s13567-019-0660-0 (PMC6549377; doi:10.1186/s13567-019-0660-0)
Supplement: Supplementary file 3 — Additional file 3. Differentially secreted proteins of the wild-type strain Yb2 and complementation strain cYb2ΔgldM. [file 13567_2019_660_MOESM3_ESM.docx]

**Additional file 3 The differential secretory proteins of wild-type strain Yb2 and complementation strain cYb2Δ*gldM*.**

| **Locus**  **tag** | **Predicted**  **protein** | **Peptides^1^** | **Unique peptides^2^** | **Sequence coverage [%]** | **MW^3^ [kDa]** | **LFQ^4^ intensity**  **of Yb2(A)** | **LFQ intensity**  **of cYb2*ΔgldM* (B)** | | **B/A** | | | |
| --- | --- | --- | --- | --- | --- | --- | --- | --- | --- | --- | --- | --- |
|  |  |  |  |  |  | **Mean** | | **Mean** | **Ratio** | ***P* value** | |  |
| AS87_RS08210  AS87_RS05440 | Uncharacterized protein Ragb/susd domain protein | 5  25 | 5  25 | 29.5  57.3 | 27.513  54.299 | 21579766.67  35154333333 | 38033000  8338333333 | | 4.509548  4.21599  0.445765  0.413494  0.390068  0.377874  0.368453  0.35099  0.300404 | | 0.038803  0.00037  0.007883  0.28311  0.01126  0.02075  0.028411  0.01619  0.031632 | |
| AS87_RS02025 | Uncharacterized protein | 1 | 1 | 2.9 | 35.731 | 7038600 | 6925400 | |  |  |  |  |
| AS87_RS08990  AS87_RS01755  AS87_RS01625  AS87_RS04945  AS87_RS07255  AS87_RS07980 | Tonb-dependent receptor plug  Uncharacterized protein  Thioredoxin  Uncharacterized protein  Uncharacterized protein  Uncharacterized protein | 25  2  2  3  3  6 | 25  2  2  3  3  6 | 24  12.4  9.3  9.5  8.9  13.8 | 118.3  15.112  21.261  31.962  30.957  47.958 | 504030000  32608000  109261333.3  14907000  86212333.33  347393333.3 | 208413333.3  12719333.33  74584000  5492533.333  30259666.67  104358333.3 | |  |  |  |  |
| AS87_RS07975 | Uncharacterized protein | 2 | 2 | 7.9 | 23.802 | 99544333.33 | 26563333.33 | | 0.266849 | | 0.044305 | |
| AS87_RS03515  AS87_RS06320  AS87_RS05130  AS87_RS04975  AS87_RS00115  AS87_RS05135  AS87_RS04955  AS87_RS08785  AS87_RS01725  AS87_RS07090  AS87_RS05110  AS87_RS02840  AS87_RS07915  AS87_RS09205  AS87_RS06535  AS87_RS05320  AS87_RS06310  AS87_RS02220  AS87_RS01595  AS87_RS07835  AS87_RS06050  AS87_RS01130  AS87_RS03125  AS87_RS00520  AS87_RS03830  AS87_RS05910  AS87_RS04955  AS87_RS08905  AS87_RS10325  AS87_RS08345  AS87_RS09110  AS87_RS03180  AS87_RS03880  AS87_RS00035  AS87_RS07985 | Glutathione peroxidase  Accessory colonization factor AcfC  Glutamyl-tRNA reductase  Uncharacterized protein  Uncharacterized protein  Hydroxymethylbilane synthase  Uncharacterized protein  Branched-chain-amino-acid aminotransferase  Transketolase, C-terminal subunit  3-oxoacyl-[acyl-carrier-protein] synthase 2  Uncharacterized protein  Uncharacterized protein  Uncharacterized protein  Uncharacterized protein  Adenine deaminase  Response regulator containing CheY-like receiver, AAA-type ATPase, and DNA-binding domains  Uncharacterized protein  2-amino-3-ketobutyrate coenzyme A ligase  Cytidine deaminase  Uncharacterized protein  Peptidoglycan glycosyltransferase  Phosphoribosylformylglycinamidine (FGAM)  synthase, synthetase domain protein  Uncharacterized protein  Uncharacterized protein  Undecaprenyl phosphate N, N'-diacetylbacillosamine  1-phosphate transferase  DNA topoisomerase 1  Uncharacterized protein  Type I site-specific restriction-modification system,  R (Restriction) subunit-related helicase  Peptidase E  Uncharacterized protein  von willebrand factor type a  Uncharacterized protein  UDP-N-acetyl-D-mannosaminuronate dehydrogenase  Uncharacterized protein  Uncharacterized protein | 8  3  2  3  15  2  1  1  2  3  4  2  1  1  2  1  3  4  1  2  2  1  8  4  1  1  2  1  2  3  1  4  2  2  2 | 8  3  2  3  1  2  1  1  2  3  1  2  1  1  2  1  3  4  1  2  2  1  1  4  1  1  2  1  2  3  1  1  2  2  2 | 66  10.4  3.1  6.6  29.4  7.2  4.2  2.8  6.7  7.5  30.8  3.1  4.1  9.9  7.2  2.1  11.1  11.1  5  0.6  3.2  0.7  46.4  25.7  4.5  1.5  15.3  1.1  7  10.9  4.2  24.6  6.1  1  9.2 | 21.146  33.042  47.597  18.32  59.104  34.106  22.139  40.208  34.014  44.385  13.278  69.904  34.281  10.901  38.079  47.941  27.171  43.638  17.691  262.97  89.03  137.68  22.902  16.247  23.185  95.462  20.645  133.6  25.938  26.849  37.658  22.735  47.247  175.39  28.842 | 51985333.33  40832666.67  12905300  3626700  4970666.667  6888000  2935066.667  561235  2935066.667  4859933.333  6776766.667  24995333.33  18644300  13050533.33  6065000  7999000  8028300  3440850  35957666.67  92168000  3450933.333  837090  6141650  8184233.333  8317400  15958500  6071333.333  4608600  231985000  2703366.667 | 12100200  6472433.333  10359900  53523000  13275566.67  326870  16585500 | | 0.232762  0.158511  +∞  +∞  +∞  +∞  +∞  -∞  -∞  -∞  -∞  -∞  -∞  -∞  -∞  -∞  -∞  -∞  -∞  -∞  -∞  -∞  -∞  -∞  -∞  -∞  -∞  -∞  -∞  -∞  -∞  -∞  -∞  -∞  -∞ | | 0.009301  0.044953  N  N  N  N  N  N  N  N  N  N  N  N  N  N  N  N  N  N  N  N  N  N  N  N  N  N  N  N  N  N  N  N  N | |

^1^Peptide counts of the protein identified by LC-MS

^2^The specific peptide counts of the protein identified by LC-MS

^3^Mol. Weight of the protein

^4^Using the Label-free quantification method to quantify protein
